# Surgical face masks impair human face matching performance for familiar and unfamiliar faces

**DOI:** 10.1186/s41235-020-00258-x

**Published:** 2020-11-19

**Authors:** Daniel J. Carragher, Peter J. B. Hancock

**Affiliations:** Psychology, Faculty of Natural Sciences, University of Stirling, Stirling, FK9 4LA Scotland, UK

**Keywords:** Face recognition, Identity verification, Familiarity, Deep neural network, Signal detection theory

## Abstract

In response to the COVID-19 pandemic, many governments around the world now recommend, or require, that their citizens cover the lower half of their face in public. Consequently, many people now wear surgical face masks in public. We investigated whether surgical face masks affected the performance of human observers, and a state-of-the-art face recognition system, on tasks of perceptual face matching. Participants judged whether two simultaneously presented face photographs showed the same person or two different people. We superimposed images of surgical masks over the faces, creating three different mask conditions: control (no masks), mixed (one face wearing a mask), and masked (both faces wearing masks). We found that surgical face masks have a large detrimental effect on human face matching performance, and that the degree of impairment is the same regardless of whether one or both faces in each pair are masked. Surprisingly, this impairment is similar in size for both familiar and unfamiliar faces. When matching masked faces, human observers are biased to reject unfamiliar faces as “mismatches” and to accept familiar faces as “matches”. Finally, the face recognition system showed very high classification accuracy for control and masked stimuli, even though it had not been trained to recognise masked faces. However, accuracy fell markedly when one face was masked and the other was not. Our findings demonstrate that surgical face masks impair the ability of humans, and naïve face recognition systems, to perform perceptual face matching tasks. Identification decisions for masked faces should be treated with caution.

## Significance statement

In response to the global COVID-19 pandemic, many governments around the world now recommend, or require, that their citizens wear face coverings in public. The increase in the number of people wearing surgical face masks in public poses unique challenges for face recognition and identification. One such task is perceptual face matching, where an observer decides whether two simultaneously presented images show the same person or two different people. Our study shows that human performance on face matching tasks is significantly worse for faces wearing surgical masks, regardless of whether one, or both faces, are masked. Surprisingly, face masks caused a similar decrease in matching performance for familiar and unfamiliar faces. We tend to make false positive decisions when familiar faces wear masks, and false rejections when unfamiliar faces wear masks. Finally, we also show that a state-of-the-art face recognition system tended to outperform human observers on these tasks, even though it had not been trained to identify masked faces; however, accuracy still decreased when one face wore a mask and the other did not. However, we also show that not all naïve face recognition systems can accurately identify masked faces. In conclusion, our study shows that both humans and naïve face recognition systems have difficulty accurately matching faces that have been covered by surgical masks.

## Introduction

Whether crossing international borders or buying alcohol at the local store, the human face is often used to verify an individual’s identity. Yet, our ability to accurately decide whether two simultaneously presented photographs show the same person varies significantly, depending on whether the person is already known to us (Kramer et al. 2018). Matching familiar faces is very easy (Bruce et al. 2001; Clutterbuck and Johnston 2002; Jenkins et al. 2011), even under challenging conditions, such as deliberate disguise (Noyes and Jenkins 2019). On the other hand, accurately matching unfamiliar faces is surprisingly difficult (Bruce et al. 1999; Megreya and Burton 2006). Even if the two photographs are taken just minutes apart, participants make errors on approximately 20% of trials (Burton et al. 2010). Difficulty in unfamiliar face matching is found for image to image comparisons (Bruce et al. 1999; Burton et al. 2010; Megreya and Burton 2006), and when comparing an image to real person (Kemp et al. 1997; White et al. 2014a). But it is not just naïve observers who find face matching to be difficult. Passport renewal officers make a similar number of errors as untrained university students when matching unfamiliar faces (White et al. 2014b).

Although unfamiliar face matching is already error prone under near optimal conditions (Burton et al. 2010), performance deteriorates even further in less than ideal conditions (Fysh and Bindemann 2017). Minor differences between the images themselves can affect accuracy, such as whether they are presented in colour or black and white (Bobak et al. 2019), whether the distance between the individual and the camera differs in each photograph (Noyes and Jenkins 2017), or if there is degradation to the image quality (Bindemann et al. 2013). Accuracy also falls if the faces are shown from different viewpoints (Estudillo and Bindemann 2014), or under different lighting conditions (Hill and Bruce 1996). Moreover, the amount of time elapsed between the capture of the two photographs can impair matching accuracy (Megreya et al. 2013), as can even minor changes to the appearance of the individual, such as whether or not they are wearing reading glasses (Graham and Ritchie 2019; Kramer and Ritchie 2016).

In response to the global COVID-19 pandemic, the United States Centers for Disease Control and Prevention (2020) has recommended that all American citizens should cover the lower portion of their face (the nose, mouth and chin) when in public. Governments around the world have made similar recommendations, often requiring citizens to wear face coverings in public spaces or on public transport (Al Jazeera News 2020). Unsurprisingly, the number of people around the world wearing face masks in public has increased dramatically (Morning Consult 2020; YouGov 2020). Disposable surgical masks, which are typically worn by healthcare professionals, have become a popular choice of face covering for the general public. The increase in the number of people wearing face masks in public poses challenges to tasks that require face recognition and identification. Already, there are reports of crimes being committed by individuals wearing surgical face masks, presumably to disguise or hide their appearance (Babwin and Dazio 2020; Southall and Van Syckle 2020). Soon, law enforcement will likely have cases in which the only CCTV footage of the crime being committed shows the perpetrator wearing a face mask. Although we can intuit that it is harder to identify people with partially covered or occluded faces, there is a surprising lack of research about how covering the lower half of the face affects performance on perceptual face matching tasks.

The effect that occluding different internal facial features has on identification accuracy has been studied in recognition memory tasks, which differ from perceptual face matching tasks because the learned and test faces are presented sequentially. These studies have revealed that different facial features are more useful than others for identifying individuals. Often, it is the upper half of the face (Dal Martello and Maloney 2006; Davies et al. 1977; Fisher and Cox 1975), and specifically the eyes (McKelvie 1976; Roberts and Bruce 1988), that has a larger influence on face recognition accuracy than the lower face (e.g., nose, mouth, chin). Yet, there are several reasons to think that surgical face masks will impair perceptual face matching performance. Although the features of the lower face are less informative for identity decisions (Fisher and Cox 1975), several of these same memory studies show that covering the mouth still reduces recognition accuracy for faces compared to when they are learned unobstructed (Davies et al. 1977; McKelvie 1976). One possible cause of this impairment is that covering the lower half of the face might disrupt the holistic processing of the face itself (Tanaka and Farah 1993; Tanaka and Sengco 1997), since it is no longer possible for the observer to gauge the spatial relations between key facial features (Maurer et al. 2002). In more realistic paradigms, faces wearing ski-masks (Manley et al. 2019) or masks made from nylon stockings (Davies and Flin 1984; Mansour et al. 2012), when learned were remembered less accurately in the subsequent recognition test than faces learned without disguise. Most relevant to the current environment, covering the entire lower face with a bandana has also been shown to impair recognition memory for faces (Nguyen and Pezdek 2017).

To the best of our knowledge, only one previous study has investigated the effect of disguising the internal features of the face in a perceptual face matching task (Dhamecha et al. 2014); moreover, surgical face masks were one type of disguise included in the study. Dhamecha et al. (2014) reported that disguising the lower half of the face impaired human face matching performance, and that familiarity with the disguised identities lessened the impairment. However, there are several aspects of this study that limit the conclusions that can be drawn about the impairment caused by surgical face masks. First, the specific impairment caused by face masks alone was not reported because different types of disguises were grouped together for analysis (e.g., surgical masks, fake beards and moustaches). Second, each participant completed just 8 face matching trials, which featured randomly intermixed disguises (e.g., the same pair might show a face wearing a surgical mask and another face disguised with sunglasses). Finally, the personal familiarity of each participant with the 75 different models in the stimuli set was not measured; rather, familiarity was assumed because the participants and the models came from the same university department. Therefore, while Dhamecha et al. (2014) offer preliminary evidence that occluding the lower face impairs human face matching performance, many questions remain about the nature of the impairment that is specifically caused by surgical face masks.

The overarching aim of the current study was to systematically document the effect that surgical face masks have on human performance in perceptual face matching tasks. We investigated whether surgical masks impair human face matching performance, whether performance differs when one face in each pair is masked compared to when both faces are masked, and whether any impairment matching unfamiliar faces also extends to familiar faces. To address these research questions, all participants in the current study completed two face matching tasks; the short version of the Glasgow Face Matching Test (GFMT; Burton et al. 2010), and the Stirling Famous Face Matching Task (SFFMT), which we developed for the current study. In both tasks, two faces are presented simultaneously, and participants indicate whether they show the same person or two different people. Participants were randomly assigned to complete both face matching tasks in one of three possible mask conditions^1^; control (wherein neither face wore a mask), mixed (one face in each pair wore a mask) and masked (both faces wore masks). Images of surgical face masks were superimposed over the original face stimuli using photo editing software (GIMP Team 2019).

We predicted that human face matching performance^2^ would be highest in the control condition and significantly reduced in the masked condition. Interestingly, an additional decrease in performance is often reported for “incongruent” conditions (Bobak et al. 2019), such as when one face is wearing glasses and the other is not (Kramer and Ritchie 2016), suggesting that performance in the masked condition will be higher than the mixed condition. Consequently, we predicted that sensitivity (measured using *d'*; Macmillan and Creelman 2004) would be highest in the control condition, reduced in the masked condition, and lowest for the mixed condition. In the SFFMT, we expected that sensitivity would be higher for familiar than unfamiliar faces, and that surgical face masks would cause greater impairment to matching performance for unfamiliar faces than familiar faces (Noyes and Jenkins 2019). Finally, we predicted that sensitivity on the GFMT (Burton et al. 2010) would be positively correlated with sensitivity on the SFFMT for all three mask conditions.

In addition to testing human observers, we also investigated whether surgical face masks would impair the performance of a state-of-the-art face recognition system. This face recognition system is a deep neural network (DNN) that was developed by the University of Surrey, which we had access to through the FACER2VM project.^3^ Importantly, the DNN was not trained to identify masked or occluded faces, which has previously proven to be a challenging task for naïve face recognition systems (Dhamecha et al. 2014; Hung et al. 2018). Our aim in testing the DNN is to see whether any impairment to human performance would be mirrored in the performance of the naïve computer system. The DNN completed the same “one-to-one” GFMT and SFFMT tasks as our human participants, once in each mask condition (control, mixed, masked).

The DNN produces a similarity rating and a classification decision (“match” or “mismatch”) for each pair of faces that it compares. The similarity rating is a match score^4^ that can range from − 100 to 100; any score above 40 is classified as a “match”. We predicted that the similarity ratings would be influenced by mask condition, such that the ratings would be highest for the control stimuli, reduced for the masked condition, and lowest for the mixed condition, when the pairs were genuine matches (it is possible that the opposite pattern of results will occur for mismatches). Yet, after observing the performance of this same DNN in unrelated studies (Hancock et al. 2020), we also predicted that classification accuracy would remain near ceiling in all mask conditions. As such, the DNN’s accuracy should be similar to human observers for familiar faces, and superior for unfamiliar faces. Finally, we tested whether the DNN would show evidence of overfitting, which occurs when performance is better for identities that the DNN was initially trained on (prior to the current study, the DNN was trained on an image set that contained famous identities, many of which are also included in our SFFMT). Overfitting would be signalled by better performance for familiar faces than unfamiliar faces on the SFFMT.

## Method

### Sample size

We conducted a power analysis to guide our choice of sample size. We based our predicted effect size on that found by Kramer and Ritchie (2016) for the effect of reading glasses on face matching performance, which was $\eta^{2}$ = 0.13 in a one-way ANOVA with glasses condition (control, one face wearing glasses, both faces wearing glasses) as a within-participants factor. An a priori power analysis (Faul et al. 2007) indicated that a total sample of 69 participants would be required to achieve 80% power to detect an effect of $\eta_{p}^{2}$ = 0.13 with a conventional alpha of *α* = 0.05 in a one-way ANOVA with 3 levels (mask condition: control, mixed, masked). Although the power analysis indicated that we only needed to recruit 23 participants per mask condition for the GFMT, the SFFMT includes an additional within-participants factor (familiarity: familiar, unfamiliar). Without having an appropriate prior study to estimate the likely effect size for a mask condition by familiarity interaction, we chose to double our sample size to account for the additional within-participants factor. Therefore, we aimed to recruit approximately 50 participants to each mask condition.

### Participants

We recruited 201 participants from the online research platform *Prolific*. All participants were aged 18 years or older and reported living in the UK. To maintain data integrity, we applied several pre-registered exclusion criteria to the collected data prior to analysis, in the following order. Participants with incomplete data were excluded (*n* = 12), as were those who attempted to complete the experiment more than once (*n* = 17),^5^ or who reported a technical issue (*n* = 7). Participants who took less than 12 min to complete the experiment were excluded (*n* = 4), as were those who took longer than 70 min (*n* = 2).^6^ Finally, participants who made the same response on ≥ 95% of trials in the GFMT (*n* = 2) or SFFMT (*n* = 1) were excluded, as were those who accurately recognised fewer than 25% of the famous identities in the recognition test (*n* = 18).

The final sample consisted of 138 participants: 53 participants in the control condition (35 females, *M*_age_ = 31.6, SD = 8.7), 43 participants in the mixed condition (25 females, *M*_age_ = 33.2, SD = 11.7), and 42 participants in the masked condition (24 females, *M*_age_ = 30.0, SD = 8.9). This research was approved by the General University Ethics Panel at the University of Stirling. All participants gave their informed consent before starting the experiment, were debriefed on completion, and were reimbursed £3 for their time.

### Materials

#### Surgical face masks

**Fig. 1:**
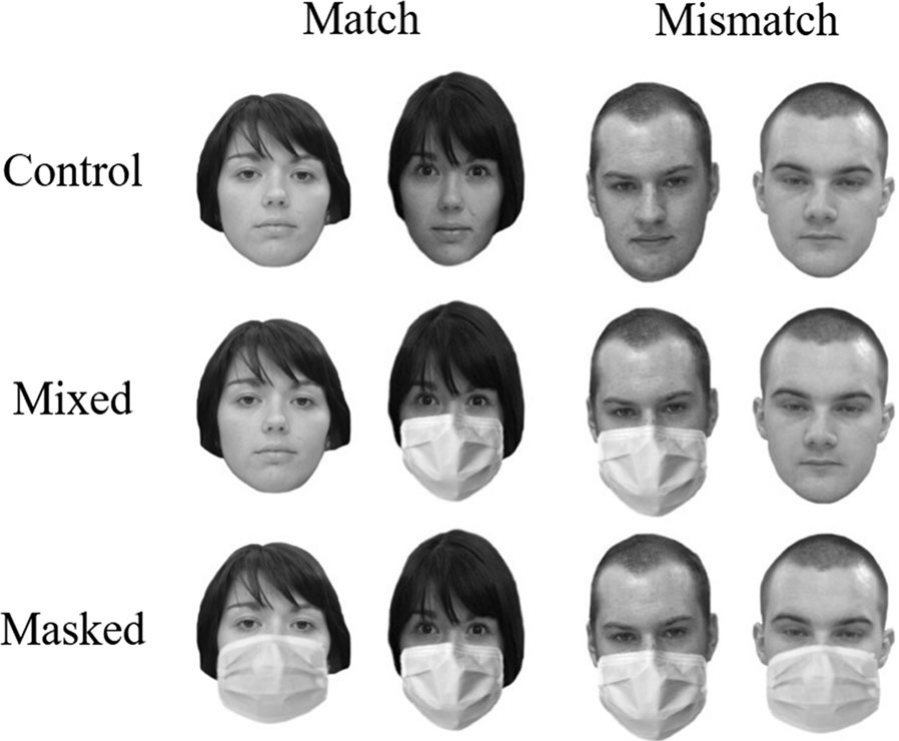
Examples of the control, mixed and masked conditions for match and mismatch trials of the GFMT [ reproduced and adapted with permission from the copyright holder]

We collected images of different surgical face masks online. The images of surgical face masks were superimposed over the original face stimuli using photo editing software (GNU Image Manipulation Program, version 2.10.8; GIMP Team 2019). Although superimposing masks over the stimuli might remove cues to facial shape that could possibly have aided matching decisions had the faces actually been wearing face masks, this approach allowed us to use the same stimuli in each mask condition, ensuring that any differences in performance could not be attributed to having used different stimuli between conditions. Nonetheless, the face masks were fitted over the stimuli so that they covered the same features of the face that a worn mask would, from the middle of the nose to below the chin (see Fig. 1). For participants in the mixed condition, the face that was masked appeared equally often on the left and right side of the image pair. In the masked condition, a different surgical mask image was randomly chosen for each face in the pair, reducing the possibility that responses would be influenced by the mask image itself.

#### Glasgow face matching test

Participants initially completed the short version of the GFMT (Burton et al. 2010), which consists of 20 match and 20 mismatch trials. The GFMT was created by taking photographs of the same person on the same day, using different cameras. Each image pair consists of one high quality image from a digital camera and one lower quality image that was extracted from a digital video recording. In the mixed condition, the mask was always placed on the lower quality image in the pair. As in the original GFMT, all stimuli were presented in greyscale, and each face image was presented at a width of 350 px. Trial order within the task was randomised.

#### Stirling famous face matching task

The SFFMT was created for the current study, to investigate whether surgical face masks would affect familiar face matching. The SFFMT consists of images of famous celebrities (familiar faces) and non-famous models (unfamiliar faces) that our laboratory has previously collected from a variety of internet sources. Models were selected as the unfamiliar stimuli because many high-quality, labelled images are available online for each model. Because these images were captured in the wild, they vary widely on many factors including image quality, lighting conditions, pose, orientation, and facial expression. In general, the images (both celebrity and model) selected for the SFFMT were high quality, well lit, and captured the face in near frontal orientation (which allowed us to place the surgical mask on the face realistically). The subjects tended to show neutral or positive facial expressions. Because the images were collected online, we have no specific information about how much time may have elapsed between capturing the two pictures of the same person. After collecting the images, we used face landmarking software developed through the FACER2VM project to locate the eyes and then scale, rotate and crop each image to show only the head and neck of each subject, with the eye positions fixed. The SFFMT was presented in colour and each face was presented at 350 × 496 px.

The SFFMT consists of 80 trials, of which 40 are familiar (famous celebrities), and 40 are unfamiliar (non-famous models). Within each familiarity condition (familiar, unfamiliar), there are 20 match and 20 mismatch trials, as there are in the GFMT (Burton et al. 2010). The SFFMT is also balanced for sex (half of all trials are female pairs). Inherently, match trials show two different images of the same identity. Mismatch trials were created by pairing two different identities that appeared visually similar (e.g., same gender, ethnicity, approximate age, hair style). Importantly, all familiar mismatch trials consist of two different celebrities that resemble each other (i.e., Jenkins et al. 2011). For these familiar mismatch trials, the face that we expected to be the less famous of the celebrity pair was always masked in the mixed condition.^7^ No identity appears in both a match and mismatch trial. All trial conditions were intermixed and randomised.

#### Recognition test

The SFFMT was followed by a recognition test, which we used to identify the familiar faces that were actually known by each participant (and to check whether any of the unfamiliar faces were recognised). All participants were presented with full unaltered faces in the recognition test, which were different images than those used in the SFFMT. Since all identities in the SFFMT were tested, the recognition test consisted of 120 trials (20 match identities & 40 mismatch identities, for familiar and unfamiliar faces). Faces were presented one at a time, in colour, at 350 × 496 px. The presentation of familiar and unfamiliar identities was intermixed and randomised.

Responses from the recognition test were used to ensure that the analysis of the SFFMT only included data from trials that participants were familiar with the famous identities (and not familiar with the unfamiliar identities). Since no participant recognised any of the unfamiliar faces, the analysis of unfamiliar faces includes data from all trials (20 match, 20 mismatch). We excluded data from familiar match trials if participants did not recognise the identity, which left an average of 14.5 match trials for each participant (control: *M* = 15.1, SD = 4.0; mixed: *M* = 14.8, SD = 4.2; masked: *M* = 13.5, SD = 4.0). We excluded data from familiar mismatch trials if participants did not recognise *either* famous identity in the pair, which also left an average of 14.5 mismatch trials for each participant (control: *M* = 14.8, SD = 4.1; mixed: *M* = 14.7, SD = 4.1; masked: *M* = 13.8, SD = 4.2).

### Procedure

The experiment was presented online using *Qualtrics* survey software. The two face matching tasks had the same trial procedure. Two faces were presented onscreen simultaneously, slightly offset to the left and right of screen centre. Participants were asked to decide whether the pair showed the same person or two different people. Responses were made by mouse click on response buttons that were labelled “same” or “different”. Each pair of faces remained on screen until response, after which the next pair was presented immediately. Participants always completed the GFMT before the SFFMT, which allowed us to measure the correlation in performance on the two tasks (an instruction screen separated the two tasks). Following the SFFMT, participants started the recognition test. A single image was presented to the centre of the screen. First, participants made a “yes/no” response to the question “do you know this person?”. If “yes” was selected, participants were asked “who is this person?”, and could respond by typing the name of the person, or by providing identifying information to show their familiarity (e.g., the name of a character they play in a TV show, or a movie they appear in). If the participant selected “no”, the next trial began. The experiment took an average of 29 min to complete (SD = 12.5).

### Analysis

We used hits (correctly responding “same” on a match trial) and false alarms (incorrectly responding “same” on a mismatch trial) to calculate the signal detection measures *d*′ (“*d*-prime”; sensitivity) and criterion *c* (response bias) for each participant (Macmillan and Creelman 2004; Stanislaw and Todorov 1999). Sensitivity is a measure of an individual’s ability to correctly distinguish true matches from true mismatches (with greater values indicating better performance), while *c* shows whether an individual had a bias towards responding “same” or “different” (Macmillan and Creelman 2004). To simplify our results section, we describe all statistical tests in the paragraph below.

To examine human performance on the GFMT, we conducted a one-way analysis of variance (ANOVA) for each measure of performance (*d*′, *c*), with mask condition (3: control, mixed, masked) as a between-participants factor. For human performance on the SFFMT, we conducted separate mixed-model ANOVAs for each measure of performance, with mask condition as a between-participants factor and familiarity (2: familiar, unfamiliar) as a within-participants factor. For the DNN, descriptive statistics are presented in figures for classification accuracy on the GFMT and SFFMT. Because there is only one DNN, we conducted item-analyses to investigate the effect that mask condition had on the similarity ratings given to each pair. For the GFMT, we conducted separate one-way ANOVAs for match and mismatch trials with mask condition as a repeated factor. For the SFFMT, we conducted separate mixed-model ANOVAs for match and mismatch trials, with mask condition as a repeated factor and familiarity as a between-item factor.

For the sake of brevity, the descriptive and inferential statistics from each analysis are reported in tables throughout the results section. Written summaries of the data in text are supported by significant inferential tests. Any violation to the assumption of homogeneity has been corrected by reporting Welch’s F test, while violations to the assumption of sphericity have been corrected by reporting Greenhouse–Geisser adjusted degrees of freedom. Simple main effects analyses are used to interpret all significant interactions, and all post hoc *t* tests have been adjusted for multiple comparisons using Bonferroni corrections (denoted by “*p*_*bonf*_”). Cohen’s *d* is the effect size reported for all comparisons between means. All analyses were conducted in JASP (Version 0.11.1.0; JASP Team 2019).

### Data availability

Our aims, hypotheses, design and analyses were pre-registered prior to data collection on the Open Science Framework [https://osf.io/p3rbe]. The datasets generated and analysed in the current study are available in the OSF repository [https://osf.io/n5hr7/]. A preprint of this work is maintained on *PsyArXiv* [https://psyarxiv.com/n9mt5].

## Results

### Human participants

#### Glasgow face matching test

**Fig. 2:**
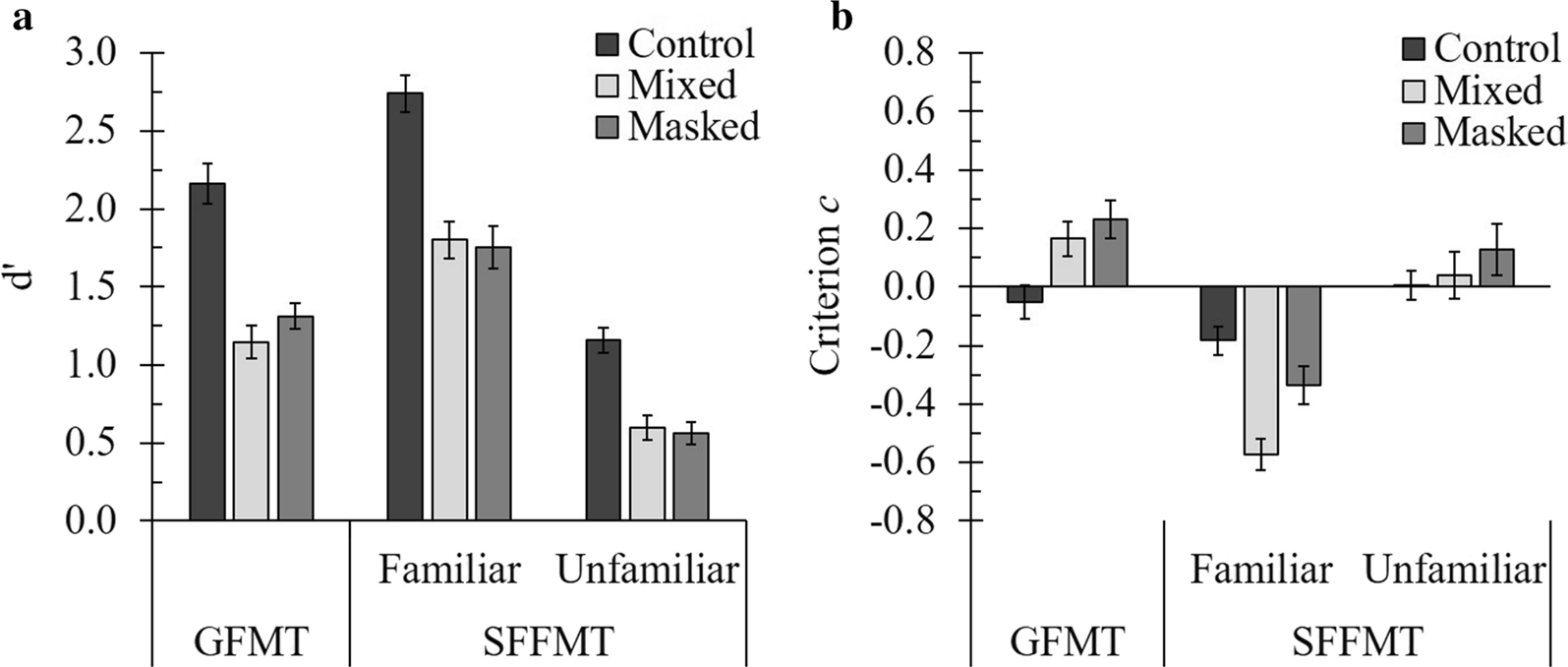
**a** Sensitivity (*d*′) and **b** response bias (criterion *c*) on the GFMT and the SFFMT (plotted separately for familiar and unfamiliar faces). Positive criterion *c* values indicate a conservative response bias (inclined to say ‘mismatch’), while negative values indicate a liberal bias. All error bars show the standard error of the mean (SEM)

The descriptive statistics for *d*′ and *c* in each mask condition are reported in Table 1. Surgical face masks had a significant effect on both sensitivity and response bias (see Table 2). Sensitivity was higher for participants in the control condition compared to those in the mixed and masked conditions (see Fig. 2a). Participants in the control condition also showed a smaller response bias than those in the mixed and masked conditions (see Fig. 2b). Response bias did not differ from chance for the control condition, whereas participants in the mixed and masked conditions displayed a conservative response bias (i.e., a bias to declare mismatches; see Table 3). The mixed and masked conditions did not differ from each other on either *d*′ or *c* (see Table 2).

**Table 1:** Descriptive statistics [mean(SD)] for measures of human performance (*d*′, *c*) on the GFMT and the SFFMT

	*d*′			*c*		
	Control	Mixed	Masked	Control	Mixed	Masked
GFMT	2.16 (0.94)	1.15 (0.70)	1.31 (0.54)	− 0.05 (0.41)	0.16 (0.39)	0.23 (0.41)
SFFMT						
Unfamiliar	1.16 (0.58)	0.60 (0.52)	0.56 (0.47)	0.01 (0.36)	0.04 (0.53)	0.13 (0.57)
Familiar	2.74 (0.87)	1.80 (0.77)	1.75 (0.89)	− 0.18 (0.35)	− 0.57 (0.36)	− 0.34 (0.42)

**Table 2:** Separate ANOVA and post hoc analyses for measures of human performance (*d*′, *c*) on the GFMT

	*d*′				*c*			
ANOVA	*F*(2, 88.89) = 20.65, *p* < .001, $\eta_{p}^{2}$ = .27				*F*(2, 135) = 6.37, *p* = .002, $\eta_{p}^{2}$ = .09			
	*t*	95% CI	*p*_*bonf*_	*d*	*t*	95% CI	*p*_*bonf*_	*d*
Control-Mixed	6.51	0.65, 1.39	< .001*	1.21	− 2.58	− 0.41, − 0.02	.033*	− 0.53
Control-Masked	5.43	0.48, 1.23	< .001*	1.08	− 3.36	− 0.48, − 0.08	.003*	− 0.68
Mixed-Masked	− 0.99	− 0.55, 0.23	.972	− 0.26	− 0.76	− 0.28, 0.14	.999	− 0.17

*Identifies statistically significant *t* tests

**Table 3:** One sample *t* tests comparing the response bias in each mask condition with 0, reported separately for the GFMT and the SFMT

	Familiar				Unfamiliar			
	*t*	95% CI	*p*	*d*	*t*	95% CI	*p*	*d*
GFMT								
Control					− 0.90	− 0.17, 0.06	.375	− 0.12
Mixed					2.76	0.04, 0.28	.009*	0.42
Masked					3.63	0.10, 0.36	< .001*	0.56
SFFMT								
Control	− 3.85	− 0.28, − 0.09	< .001*	− 0.53	0.11	− 0.09, 0.11	.916	0.02
Mixed	− 10.58	− 0.68, − 0.46	< .001*	− 1.61	0.48	− 0.13, 0.20	.637	0.07
Masked	− 5.22	− 0.47, − 0.21	< .001*	− 0.81	1.45	− 0.05, 0.31	.154	0.22

*Identifies statistically significant *t* tests

#### Stirling famous face matching task

For both measures of sensitivity and response bias, the main effects of familiarity and mask condition were significant, as was the interaction between the two factors (see Table 4). We begin by summarising the simple main effects for each measure (see Table 5).

**Table 4:** Separate repeated-measures ANOVAs for measures of human performance (d′, c) on the SFMT

	*d*′	*c*
Familiarity	*F*(1, 135) = 368.24, *p* < .001, $\eta_{p}^{2}$ = .73	*F*(1, 135) = 104.06, *p* < .001, $\eta_{p}^{2}$ = .44
Mask condition	*F*(2, 135) = 29.01, *p* < .001, $\eta_{p}^{2}$ = .30	*F*(2, 135) = 3.38, *p* = .037, $\eta_{p}^{2}$ = .05
Interaction	*F*(2, 135) = 3.69, *p* = .027, $\eta_{p}^{2}$ = .05	*F*(2, 135) = 9.56, *p* < .001, $\eta_{p}^{2}$ = .12

**Table 5:** Simple main effects (SME) analyses for the effect of mask condition on face familiarity, for measures of human performance (*d*′, *c*) on the SFFMT

	Familiar faces				Unfamiliar faces			
*d*′								
SME	*F*(2, 135) = 21.12, *p* < .001, $\eta_{p}^{2}$ = .24				*F*(2, 135) = 19.79, *p* < .001, $\eta_{p}^{2}$ = .23			
	*t*	95% CI	*p*_*bonf*_	*d*	*t*	95% CI	*p*_*bonf*_	*d*
Control-Mixed	5.39	0.53, 1.35	< .001*	1.13	5.19	0.31, 0.82	< .001*	1.02
Control-Masked	5.65	0.57, 1.40	< .001*	1.13	5.50	0.34, 0.86	< .001*	1.12
Mixed-Masked	0.28	− 0.39, 0.49	.999	0.06	0.32	− 0.23, 0.31	.999	0.08

*c*								
SME	*F*(2, 135) = 13.07, *p* < .001, $\eta_{p}^{2}$ = .16				*F*(2, 135) = 0.77, *p* = .464, $\eta_{p}^{2}$ = .01			
	*t*	95% CI	*p*_*bonf*_	*d*	*t*	95% CI	*p*_*bonf*_	*d*
Control-Mixed	5.10	0.21, 0.57	< .001*	1.11				
Control-Masked	1.97	− 0.03, 0.33	.153	0.40				
Mixed-Masked	− 2.95	− 0.43, − 0.05	.011*	− 0.62				

*Identifies statistically significant *t *tests

##### Sensitivity

Face masks had a slightly stronger effect on sensitivity for familiar than unfamiliar faces; however, the pattern of results was the same for both conditions. Sensitivity was significantly higher in the control condition compared to both the mixed and masked conditions, which did not differ from each other. Examination of the effect sizes for these comparisons in Table 5 shows that face masks cause a near identical degree of impairment when matching familiar and unfamiliar faces.

##### Response bias

Face masks only affected response bias for familiar faces. While all mask conditions showed a liberal response bias to familiar faces (i.e., a bias to declare a match), this bias was significantly larger in the mixed condition than in the control and masked conditions, which did not differ from each other (see Table 5). Response bias towards unfamiliar faces did not differ from chance in any mask condition (see Table 3).

#### Correlation between the GFMT and the SFFMT

Sensitivity on the GFMT was positively correlated with sensitivity for the familiar and unfamiliar faces of the SFFMT for participants in the control (familiar: *r* = 0.60, *p* < 0.001; unfamiliar: *r* = 0.55, *p* < 0.001) and mixed conditions (familiar: *r* = 0.44, *p* = 0.003; unfamiliar: *r* = 0.49, *p* < 0.001). For participants in the masked condition, sensitivity on the GFMT was correlated with sensitivity for the familiar (*r* = 0.44, *p* = 0.003) but not unfamiliar faces (*r* = 0.23, *p* = 0.137) of the SFFMT.

### Deep neural network

#### Classification accuracy

**Fig. 3:**
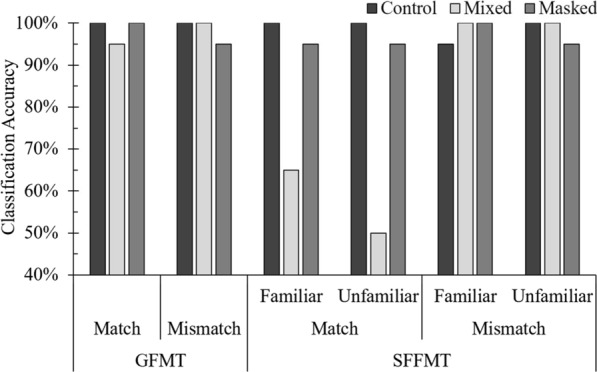
Classification accuracy of the DNN, shown as the percentage correct of the 20 trials in each condition, plotted separately for the match and mismatch trials of the GFMT and the SFFMT

The DNN showed very high accuracy on the GFMT, correctly classifying all pairs in the control condition, and making just one error for the mixed and masked stimuli (see Fig. 3). Accuracy was similarly high for the control and masked conditions of the SFFMT. However, accuracy dropped markedly for match trials in the mixed condition of the SFFMT, indicating that the DNN was more likely to reject pairs as mismatches when only one face was wearing a mask.

#### Similarity ratings

**Fig. 4:**
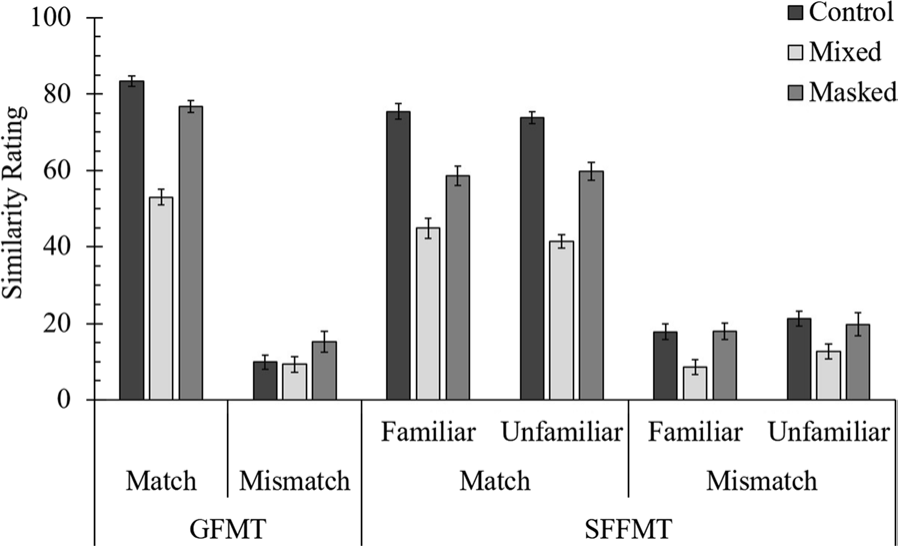
Similarity ratings from the DNN, plotted separately for the match and mismatch trials of the GFMT and the SFFMT. Error bars show SEM

The descriptive statistics for the similarity ratings given by the DNN for the GFMT and the SFFMT are reported in Table 6. Face masks had the same effect on the similarity ratings given for match trials in the GFMT and the SFFMT (see Table 7). Similarity ratings were higher in the control condition than the masked condition, which were higher than the mixed condition (see Fig. 4). Mask condition also influenced the similarity ratings for mismatched pairs in the SFFMT, with lower ratings given to the mixed condition than to the control and masked conditions, which did not differ from each other. Mask condition did not affect similarity ratings for mismatched pairs in the GFMT.

**Table 6:** Descriptive statistics [means(SD)] for the similarity ratings given by the DNN for the match and mismatch trials of the GFMT and the SFFMT

	Match			Mismatch		
	Control	Mixed	Masked	Control	Mixed	Masked
GFMT	83.35 (5.85)	52.95 (9.11)	76.75 (7.03)	9.85 (8.25)	9.30 (9.21)	15.20 (12.25)
SFFMT						
Unfamiliar	73.75 (6.90)	41.35 (8.11)	59.80 (10.68)	21.20 (8.72)	12.65 (8.46)	19.80 (13.70)
Familiar	75.45 (9.06)	44.85 (11.98)	58.60 (11.58)	17.80 (9.31)	8.65 (8.62)	17.95 (9.98)

**Table 7:** Separate item-analysis ANOVAs and post hoc analyses for the similarity ratings given by the DNN for match and mismatch trials of the GFMT and the SFFMT

	Match pairs				Mismatch pairs			
GFMT								
Mask condition	*F*(2, 57) = 92.03, *p* < .001, $\eta_{p}^{2}$ = .76				*F*(2, 57) = 2.10, *p* = .131, $\eta_{p}^{2}$ = .07			
	*t*	95% CI	*p*_*bonf*_	*d*	*t*	95% CI	*p*_*bonf*_	*d*
Control-Mixed	12.90	24.73, 36.07	< .001*	3.97				
Control-Masked	2.80	0.93, 12.27	.021*	1.02				
Mixed-Masked	− 10.10	− 29.47, − 18.13	< .001*	− 2.93				

SFFMT								
**Mask condition**	***F*(2, 76) = 194.39, *p < .001,*** $\varvec{\eta_{p}^{2}}$** = .84**				***F*(2, 76) = 19.76, *p* < .001, **$\varvec{\eta_{p}^{2}}$** = .34**			
**Familiarity**	***F*(1, 38) = 0.28, *p* = .601, **$\varvec{\eta_{p}^{2}}$** = .01**				***F*(1, 38) = 1.43, *p* = .239, **$\varvec{\eta_{p}^{2}}$** = .04**			
** Interaction**	***F*(2, 76) = 1.10, *p* = .338, **$\varvec{\eta_{p}^{2}}$** = .03**				***F*(2, 76) = 0.25, *p* = .780, **$\varvec{\eta_{p}^{2}}$** = .01**			

Mask condition	*t*	95% CI	*p*_*bonf*_	*d*	*t*	95% CI	*p*_*bonf*_	*d*
Control-Mixed	19.72	27.59, 35.41	< .001*	3.12	5.63	5.00, 12.70	< .001*	0.89
Control-Masked	9.64	11.49, 19.31	< .001*	1.52	0.40	− 3.22, 4.47	.999	0.06
Mixed-Masked	− 10.08	− 20.01, − 12.19	< .001*	− 1.59	− 5.23	− 12.07, − 4.38	< .001*	0.83

*Identifies statistically significant *t* tests

Neither the main effect of familiarity, nor the interaction between familiarity and mask condition, were significant for the match or mismatch trials in the SFFMT. Because the DNN’s performance did not differ between the unfamiliar faces and the famous identities that were included in its training set (prior to this research), our results offer no indication of overfitting by the DNN.

## General discussion

### Human performance

These results clearly show that surgical face masks significantly impair human face matching performance. Our analysis of *d*′, a measure that accounts for performance on match and mismatch trials that is unaffected by response biases (Stanislaw and Todorov 1999), revealed an identical pattern of results in both face matching tasks. Compared to control, sensitivity was significantly reduced in the mixed and masked conditions, which did not differ from each other. The differences in sensitivity between the control condition and either mask condition were large and consistent; the effect sizes from all 6 comparisons range from *d* = 1.02 to *d* = 1.21. These results demonstrate that the impairment to human face matching ability is the same, regardless of whether one, or both, faces in the pair are masked.

As predicted, human observers showed greater sensitivity when matching familiar faces than unfamiliar faces (Megreya and Burton 2007). Moreover, sensitivity for familiar faces in both mask conditions (mixed, masked) remained higher than that shown by the control condition for the unfamiliar faces of the SFFMT, demonstrating that matching masked familiar faces remains a relatively easy task. Yet, in contrast to our predictions, the interaction between mask condition and familiarity indicated that face masks caused greater impairment to familiar faces than unfamiliar faces. This finding was particularly surprising, because familiar face matching is typically robust to the types of disruptions that impair performance for unfamiliar faces (e.g., Noyes and Jenkins 2019). Although this result might signal a true difference in the effect that face masks have on familiar and unfamiliar faces, the small effect size of this interaction, coupled with the large effect that mask condition had on both familiar and unfamiliar faces, suggests that this result is most likely the product of the different baseline performance in the two conditions. Higher baseline sensitivity for familiar faces means that surgical face masks can cause a greater decrease in performance, whereas any impairment to lower sensitivity for unfamiliar faces is likely to be limited by floor effects. Our interpretation of this significant interaction as inconsequential is consistent with the effect sizes for the post hoc comparisons in Table 5, which show that the differences in sensitivity between the control, mixed, and masked conditions are nearly identical for familiar and unfamiliar faces. Together, these results clearly show that human face matching performance for both familiar and unfamiliar faces is impaired by surgical face masks.

Surgical face masks also had a significant effect on response bias in both face matching tasks. In the GFMT, participants in the mixed and masked conditions showed a bias to declare pairs as “mismatches”, whereas those in the control condition displayed no bias. Conversely, all conditions displayed a liberal response bias to the familiar faces in the SFFMT, which was exacerbated for participants in the mixed condition. Although these liberal response biases were somewhat surprising, because observers are good at matching familiar faces, they can likely be explained by the design of the SFFMT. First, the liberal response bias shown by the control condition might indicate that the participants generally did not appropriately consider the possibility that some celebrities might have look-a-likes; instead, participants might simply have responded “same” as soon as they recognised one of the celebrities in a mismatched pair. Second, for participants in the mixed condition, it was the least famous identity in the mismatch pair that was always masked (see Method). Because participants likely recognised the face that wasn’t masked, it is possible that they were able to compare all stored mental representations of the famous unmasked identity to the masked face on screen. Under these conditions, the mental comparison is no longer “do these faces belong to the same person?”, but rather “could that be Leonardo DiCaprio wearing a face mask?”. Comparisons to previously encountered exemplars are not possible for unfamiliar faces, because the observer has no other exemplars in mind (Hancock et al. 2000; Kramer et al. 2018). Regardless of whether the design of the SFFMT contributed to these biases, our findings suggest that observers are more tolerant to additional uncertainty or variance in appearance if they are matching a known identity to a masked face. When taken together, our results show that human observers are liable to make false positive decisions for familiar faces, and false rejections for unfamiliar faces, when the faces are covered by surgical masks.

### Task validity

The GFMT is a validated measure of face matching ability with established performance benchmarks (Burton et al. 2010). The overall accuracy (average of accuracy for match and mismatch trials) shown by our control condition (*M* = 82.4%, SD = 11.4) was very similar to that originally reported by Burton and colleagues (*M* = 81.3%, SD = 9.7), showing that our online sample performed as expected (see Additional file 1 for analysis of human accuracy). Moreover, although we created the SFFMT for the current study, sensitivity for both familiar and unfamiliar faces was positively correlated with sensitivity on the GFMT, suggesting that our new SFFMT is likely measuring the same face matching abilities as the GFMT (Burton et al. 2010).

### DNN performance

Overall, the DNN performed remarkably well on the GFMT and SFFMT, making no more than one classification error in any one stimulus condition for control or masked stimuli. The DNN showed similarly high accuracy for the mixed condition on the GFMT, and for the mismatched trials of the SFFMT. This level of accuracy exceeds average human performance, and is equivalent to the most sensitive human observers. However, the DNN’s accuracy fell for match pairs in the mixed condition of the SFFMT, suggesting a tendency to declare mismatches when one face was masked and the other was not. The cause of this impairment can be seen in the DNN’s similarity ratings. For all match trials, the mixed condition was rated to be less similar than the control or masked conditions. In the SFFMT, the average similarity rating for the mixed condition was just above 40, which is the threshold that this DNN uses to declare a “match”. Interestingly, the DNN only made one error for the equivalent condition in the GFMT. This discrepancy can be explained by the different difficulty of the GFMT and SFFMT; although the similarity ratings also fell for the GFMT’s mixed condition, they remained well above the threshold, preserving classification accuracy.

The high classification accuracy of the DNN was somewhat surprising, because the system was not trained to recognise masked faces, which is typically a challenging task for face recognition systems (Hung et al. 2018). Because the DNN is a black-box system, we can only infer how it processes masked faces by looking at the similarity ratings for each condition. If the DNN was actively matching face masks to other face masks, the similarity ratings for the masked condition would likely be higher than those for the control condition, which was not the case. Instead, the face masks appear to affect the performance of this DNN because they prevent it from locating the facial landmarks that it uses to compare faces (e.g., a nose, mouth, or jaw). If this inference is correct, our findings indicate that this DNN is still able to extract enough information from the top half of a masked face to perform accurate identifications. Regardless of how the DNN treats face masks, they still have the potential to interfere with classification accuracy. Unlike human observers, who can intuitively adjust their internal response threshold (criterion) for masked faces, the DNN is programmed to use a single threshold that is based on similarity ratings; once the threshold is passed, a match is declared. Because surgical face masks interfere with these similarity ratings, the thresholds used by naïve systems must be carefully examined and calibrated before they are used to match masked faces.

Overall, the ability of the DNN to match faces occluded by surgical masks was equivalent to, or exceeded, that of human observers. In Additional file 1, we investigated whether the performance of this research DNN is typical of three commercially available face recognition systems (which, to our knowledge, had also not been trained to match masked or occluded faces). Briefly, we find that the performance of one commercially available system is comparable to the research DNN, but that surgical face masks significantly impair the performance of the other two systems (one DNN often fails to realise that faces wearing masks are human faces, while the other DNN actively matches the face masks between images resulting in exceedingly high numbers of false positive classifications). Therefore, while it appears that some naïve face recognition systems might be able to recognise masked faces, other systems cannot. Clearly, extensive validation is necessary for any face recognition system that is used to identify masked faces, and particularly for those that were not trained to do so. Our findings are consistent with those of the recently published report from the National Institute of Standards and Technology regarding the performance of other naïve facial verification algorithms on a matching task with masked faces (Ngan et al. 2020). 

### Future directions

Our study is the first to demonstrate that surgical face masks significantly impair human face matching performance for both familiar and unfamiliar faces, and that the degree of this impairment is similar whether one or both faces in each pair are masked. Despite these advances, many questions remain unanswered. First and foremost, our data do not offer an insight into why face masks impair face matching ability. One possibility is simply that face masks obscure a large area of the face that includes features that are informative for performing identification tasks. However, this possibility seems unlikely since previous research has shown that the mouth and nose are less useful for identification than the features of the upper face (Davies et al. 1977; Fisher and Cox 1975; McKelvie 1976). Alternatively, the impairment might occur because the face mask disrupts the ability of the observer to engage in the holistic processing that is used in face perception (Maurer et al. 2002; Tanaka and Farah 1993). A very recent pre-print provides support for this notion, reporting that face masks do interfere with holistic processing (Freud et al. Under Review). Indeed, it is also possible that due to holistic processing, adding a mask to a face may alter the apparent appearance of the top half of the face, just as changing the identity of the bottom half does (Young et al. 1987). An experiment that compares matching performance for faces that have the lower half of the face removed entirely (i.e., Calder et al. 2000), compared to those wearing a face mask, would indicate whether this impairment is due to the absence of facial features that carry identity information or to the encoding of the mask itself (either voluntarily or involuntarily).

When one considers the possibility that face masks are likely to be worn in public for the foreseeable future, additional research would be well directed towards investigating whether the impairment they cause to face matching performance can be reduced. Previous research has had some success in using specific instructions to improve face matching performance, by encouraging individuals to focus on specific facial features (Megreya and Bindemann 2018). Perhaps instructing observers to focus on the unobscured features of a masked face (e.g., the eyes, eyebrows), or even specifically to ignore the face mask, will improve matching performance. Alternatively, providing corrective feedback for matching decisions appears to offer some benefit to face matching performance (Alenezi and Bindemann 2013; White et al. 2014a), while there is also mixed support for the efficacy of some training paradigms (Towler et al. 2019, 2014). Identifying methods to improve human face matching abilities for masked faces will reduce the occurrence of false positive or false negative identification decisions in future. Finally, one might consider whether prolonged exposure to people wearing face masks in public will improve face matching performance over time (e.g., as in the “headscarf effect”; Megreya and Bindemann 2009).

Finally, future research might also consider whether there are individual differences in matching performance for masked faces. Individual face recognition and matching abilities vary widely, from the marked impairments that are seen in individuals with prosopagnosia (Palermo et al. 2011; Susilo and Duchaine 2013), to the exceptional face recognition and matching performance that is typical of “super-recognisers” (Bobak et al. 2016a, b; Russell et al. 2009). The between-subjects design of the current study means that we are unable to compare the matching performance of the same individual participants across mask conditions. A very recent investigation of individual differences in masked face matching ability found that super-recognisers outperformed regular individuals on a face matching task when one face was wearing a face mask and the other was not (equivalent to our mixed condition; Noyes et al. Under Review). Moreover, the authors reported that performance on the original short version of the GFMT was positivity correlated with performance on the masked face matching task with a moderate effect size. Interestingly, although the super-recognisers ultimately outperformed the control participants, they appeared to experience the same degree of impairment to their matching performance when the faces were masked as did the control participants (Noyes et al. Under Review). When considered alongside our finding that a similar size impairment occurs for familiar and unfamiliar faces, this finding from Noyes et al. (Under Review) hints at the possibility that face masks might cause a relatively consistent impairment to face matching performance, regardless of the attributes of the faces or the abilities of the observer.

One limitation to the current study is that our stimuli were not actually wearing face masks (the face masks were superimposed over each image using photo editing software). Matching images of people who are actually wearing face masks might be a slightly easier task, since it is possible that some information about facial shape might be preserved by a surgical mask that is worn. However, we note that surgical face masks are not generally designed to be form-fitting, which raises questions about whether enough shape information would be preserved to aid matching decisions. One advantage to our methodology is that the same underlying face images were shown in each mask condition. Using images of people actually wearing masks would necessarily involve using different sets of images between mask conditions, which would leave open the possibility that any difference in face matching performance might be due to stimulus differences, rather than the face masks themselves (Jenkins et al. 2011). Another benefit to our approach is that we were able to create masked versions of famous faces, which allowed us to test the effect of masking familiar faces without needing to create a personalised set of familiar stimuli for each participant. Nonetheless, future researchers might choose to replicate the current study using images of people wearing face masks (see Noyes et al. Under Review).

## Conclusion

Covering the lower half of the face with a surgical face mask clearly has a large detrimental effect on human performance in perceptual face matching tasks. Interestingly, the degree of impairment is similar whether one or both faces in the pair are wearing masks. Surprisingly, face masks have the same detrimental effect on the matching of familiar faces as they do unfamiliar faces. Masking familiar faces can bias participants to declare matches, whereas masking unfamiliar faces causes a bias towards declaring mismatches. The performance of the research DNN matched or exceeded human performance in all mask conditions, which raises the possibility that some naïve face recognition systems might be able to accurately match faces wearing surgical masks (however, see Additional file 1). In light of these findings, future efforts would be well directed towards creating transparent face coverings that can reduce the spread of disease, while still allowing the identification of the individual underneath.

## Supplementary information

**Additional file 1:** Additional analysis of human accuracy data, and reports the performance of three commercial face recognition systems on the GFMT and SFFMT.

## Data Availability

The datasets generated and analysed in the current study are available in the OSF repository [https://osf.io/n5hr7/].

## Abbreviations

DNN: Deep neural network
GFMT: Glasgow face matching test
SFFMT: Stirling famous face matching task

## Footnotes

1 Mask condition was deliberately varied between subjects to prevent participants from adopting strategic response behaviours, such as taking less care on trials that showed faces covered by masks.

2 Here we use “performance” to mean both sensitivity (*d'*) and accuracy. Sensitivity is the focus of the main text. A full analysis of accuracy (for match and mismatch trials) is reported in Additional file 1.

3 A commercial version of the research DNN also exists (www.sensusfuturis.com).

4 The similarity score is given by 100 times the cosine of the angle between the 512 element vectors at the output of the DNN for each face.

5 A technical issue allowed some participants to access the experiment more than once. Consequently, we screened all collected data for multiple submissions from the same participant. Regardless of final completion status, all data were excluded from participants who accessed the experiment more than once and started the face matching tasks on more than one occasion.

6 After realising that we had underestimated the variability in completion time among real participants, we revised our pre-registered exclusion criteria for duration from a minimum of 17 min and a maximum of 45 min to 12 and 70 min, respectively. This revision was made prior to analysis and allowed us to keep data from 30 additional participants. Importantly, these additional data passed all other exclusion criteria.

7 On May 5, 2020, we collected ratings of “fame” from the polling website YouGov (https://yougov.co.uk/topics/overview/ratings), to gauge which celebrity was likely be the most famous in each familiar mismatch pair. This “fame” metric is based on the percentage of respondents, from a nationally representative (UK) sample of 6,000–6,500 participants, who “had heard of” each celebrity. We used responses from millennial respondents (those born between 1981 and 1996), as they would be closest in age to most participants in the current study. The least famous identity in each pair was then fitted with a mask for the mixed condition.

## Open practices statement

The datasets generated and analysed in the current study are available in the OSF repository [https://osf.io/n5hr7/]. Our aims, hypotheses, design, and analyses were pre-registered prior to data collection on the OSF [https://osf.io/p3rbe].
