# Supplementary material for: Surgical face masks impair human face matching performance for familiar and unfamiliar faces
Source: Cogn Res Princ Implic. 2020 Nov 19;5:59. doi: 10.1186/s41235-020-00258-x (PMC7673975; doi:10.1186/s41235-020-00258-x)
Supplement: Supplementary file 1 — Additional file 1: Additional analysis of human accuracy data, and reports the performance of three commercial face recognition systems on the GFMT and SFFMT. [file 41235_2020_258_MOESM1_ESM.docx]

**Surgical face masks impair human face matching performance for familiar and unfamiliar faces**

Daniel J. Carragher^1^*, Peter J. B. Hancock^1^

^1^Psychology

Faculty of Natural Sciences

University of Stirling

Scotland, United Kingdom

**** SUPPLEMENTARY MATERIALS ****

Total Word Count: 2475 approx.

*Corresponding Author:

Daniel J. Carragher

Faculty of Natural Sciences

University of Stirling

Stirling, UK, FK9 4LA

danieljcarragher@gmail.com

**Supplementary Analysis**

**1. Human Accuracy**

Much like the hypotheses outlined for *d'* in the main text, we predicted that human face matching accuracy would be highest in the control condition, reduced in the masked condition, and lowest in the mixed condition. For the SFFMT, we expected that accuracy would be higher for familiar than unfamiliar faces, and that surgical face masks would cause greater impairment to matching performance for unfamiliar faces than familiar faces (Noyes & Jenkins, 2019). Finally, we predicted that accuracy on the GFMT (Burton, White, & McNeill, 2010) would be positively correlated with accuracy on the SFFMT for all three mask conditions.

***Analysis***

We analysed accuracy for match and mismatch trials separately, because performance on match and mismatch trials is often only weakly correlated (Megreya & Burton, 2007). To examine human performance on the GFMT, we conducted a one-way analysis of variance (ANOVA) for each measure of performance (match accuracy, mismatch accuracy), with mask condition (3: control, mixed, masked) as a between-participants factor. For human performance on the SFFMT, we conducted separate mixed-model ANOVAs for each measure of performance, with mask condition as a between-participants factor and familiarity (2: familiar, unfamiliar) as a within-participants factor.

***Glasgow Face Matching Test***

Descriptive statistics for human accuracy on the GFMT and the SFFMT are given in Table S1). Surgical face masks significantly impaired accuracy for match trials, but not mismatch trials (see Table S2). For matching pairs, participants in the control condition were more accurate than those in the mixed and masked conditions, which did not differ from each other (see Figure S1).


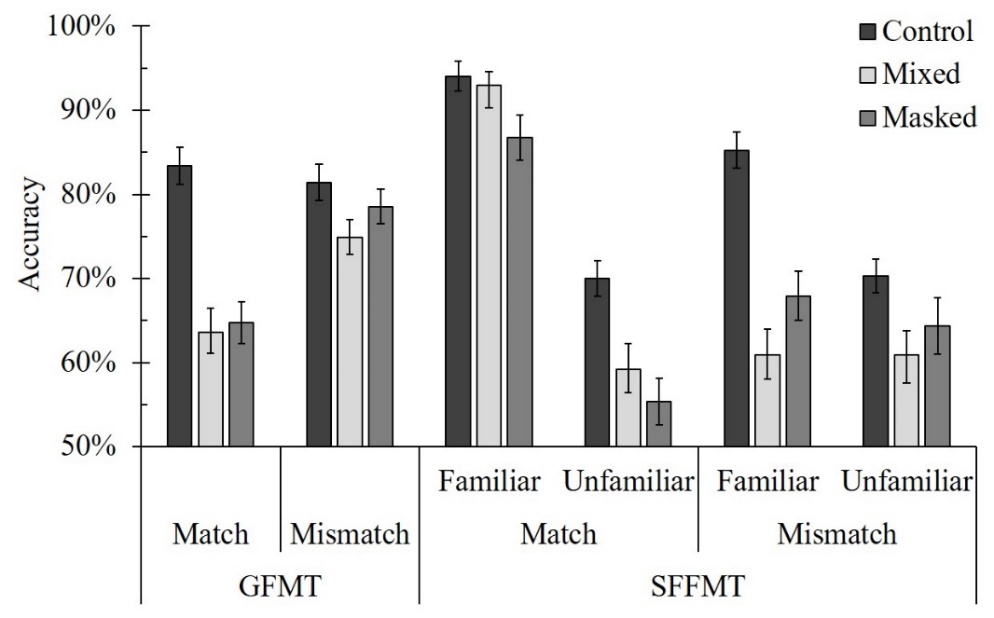


**Figure S1.** Human accuracy for match and mismatch trials, plotted separately for the GFMT and the SFFMT (plotted separately for familiar and unfamiliar faces). Error bars show the standard error of the mean (SEM).

**Table S1**

*Descriptive statistics [mean(SD)] for human accuracy (match, mismatch) on the GFMT and the SFFMT.*

|  | Match Trials | | | Mismatch Trials | | |
| --- | --- | --- | --- | --- | --- | --- |
|  | Control | Mixed | Masked | Control | Mixed | Masked |
| **GFMT** | 83.40 (15.74) | 63.61 (18.62) | 64.76 (16.04) | 81.42 (15.67) | 74.88 (14.04) | 78.57 (13.03) |
| **SFFMT** |  |  |  |  |  |  |
| Unfamiliar | 70.00 (15.57) | 59.19 (19.88) | 55.36 (17.85) | 70.28 (14.53) | 60.93 (18.91) | 64.41 (21.76) |
| Familiar | 94.07 (12.57) | 92.98 (10.74) | 86.74 (17.17) | 85.25 (15.52) | 60.93 (20.11) | 67.92 (18.85) |

**Table S2**

*Separate ANOVA and post-hoc analyses for human accuracy on the match trials and mismatch trials of the GFMT. *identifies statistically significant t-tests.*

|  | **Match Trials** | | | | **Mismatch Trials** | | | |
| --- | --- | --- | --- | --- | --- | --- | --- | --- |
| ANOVA | *F*(2, 135) = 21.47, *p* < .001, $\text{η}_{\text{p}}^{\text{2}}$ = .24 | | | | *F*(2, 135) = 2.44, *p* = .091, $\text{η}_{\text{p}}^{\text{2}}$ = .04 | | | |
|  | ***t*** | **95%CI** | ***p_bonf_*** | ***d*** |  |  |  |  |
| Control-Mixed | 5.75 | 11.63, 27.95 | < .001* | 1.16 |  |  |  |  |
| Control-Masked | 5.38 | 10.42, 26.85 | < .001* | 1.17 |  |  |  |  |
| Mixed-Masked | -0.32 | -9.78, 7.47 | .999 | -0.07 |  |  |  |  |

***Stirling Famous Face Matching Task***

The main effects of familiarity and mask condition were significant for both measures of human accuracy (match trials, mismatch trials), as was the interaction between the two factors (see Table S3). We begin by summarising the simple main effects for each measure of accuracy (see Table S4).

For match pairs, face masks caused greater impairment to unfamiliar faces than familiar faces (see Table S4); however, a similar pattern was seen for both familiarity conditions (see Figure S1). Participants in the control condition were more accurate than those in the masked condition, while the mixed and masked conditions did not differ. For unfamiliar faces only, the control condition was more accurate than the mixed condition.

Conversely, face masks caused greater impairment to familiar faces than unfamiliar faces when the pairs were true mismatches; but again, the pattern was similar for both familiarity conditions (see Table S4). Accuracy was higher in the control condition than the mixed condition, while the mixed and masked conditions did not differ. For familiar faces only, accuracy was higher in the control condition than the masked condition.

**Table S3**

*Separate repeated-measures ANOVAs for human accuracy on the match trials and mismatch trials of the SFMT.*

|  | **Match Trials** | **Mismatch Trials** |
| --- | --- | --- |
| Familiarity | *F*(1, 135) = 346.03, *p* < .001, $\text{η}_{\text{p}}^{\text{2}}$ = .72 | *F*(1, 135) = 13.45, *p* < .001, $\text{η}_{\text{p}}^{\text{2}}$ = .09 |
| Mask Condition | *F*(2, 135) = 8.82, *p* < .001, $\text{η}_{\text{p}}^{\text{2}}$ = .12 | *F*(2, 135) = 15.33, *p* < .001, $\text{η}_{\text{p}}^{\text{2}}$ = .19 |
| Interaction | *F*(2, 135) = 3.58, *p* = .031, $\text{η}_{\text{p}}^{\text{2}}$ = .05 | *F*(2, 135) = 7.76, *p* < .001, $\text{η}_{\text{p}}^{\text{2}}$ = .10 |

**Table S4**

*Simple main effects (SME) analyses for the effect of mask condition on face familiarity, for human accuracy on the match trials and mismatch trials of the SFFMT. *identifies statistically significant t-tests.*

|  | **Familiar Faces** | | | | **Unfamiliar Faces** | | | |
| --- | --- | --- | --- | --- | --- | --- | --- | --- |
| **Match Accuracy** | |  |  |  |  |  |  |  |
| SME | *F*(2, 135) = 3.74, *p* = .026, $\text{η}_{\text{p}}^{\text{2}}$ = .05 | | | | *F*(2, 135) = 8.91, *p* < .001, $\text{η}_{\text{p}}^{\text{2}}$ = .12 | | | |
|  | ***t*** | **95%CI** | ***p_bonf_*** | ***d*** | ***t*** | **95%CI** | ***p_bonf_*** | ***d*** |
| Control-Mixed | 0.39 | -5.55, 7.73 | .999 | 0.09 | 2.98 | 2.21, 19.42 | .010* | 0.61 |
| Control-Masked | 2.60 | 0.65, 14.01 | .031* | 0.50 | 4.01 | 5.98, 23.31 | < .001* | 0.88 |
| Mixed-Masked | 2.11 | -0.78, 13.25 | .111 | 0.44 | 1.00 | -5.27, 12.93 | .961 | 0.20 |
| **Mismatch Accuracy** | |  |  |  |  |  |  |  |
| SME | *F*(2, 135) = 23.35, *p* < .001, $\text{η}_{\text{p}}^{\text{2}}$ = .26 | | | | *F*(2, 135) = 3.21, *p* = .043, $\text{η}_{\text{p}}^{\text{2}}$ = .05 | | | |
|  | ***t*** | **95%CI** | ***p_bonf_*** | ***d*** | ***t*** | **95%CI** | ***p_bonf_*** | ***d*** |
| Control-Mixed | 6.56 | 15.53, 33.10 | < .001* | 1.37 | 2.49 | 0.43, 18.27 | .043* | 0.56 |
| Control-Masked | 4.64 | 8.48, 26.17 | < .001* | 1.02 | 1.55 | -3.10, 14.86 | .369 | 0.33 |
| Mixed-Masked | -1.78 | -16.28, 2.30 | .231 | -0.36 | -0.87 | -12.90, 5.95 | .999 | -0.17 |

***Correlation between GFMT and SFFMT***

Overall accuracy (average accuracy across match and mismatch trials) on the GFMT was positively correlated with overall accuracy for the familiar and unfamiliar faces in the SFFMT for all mask conditions: control (familiar: *r* = .63, *p* < .001; unfamiliar: *r* = .53, *p* < .001), mixed (familiar: *r* = .39, *p* = .009; unfamiliar: *r* = .48, *p* = .001), masked (familiar: *r* = .47, *p* = .002; unfamiliar faces: *r* = .31, *p* = .046). These correlations further support our conclusion from the main text that our new SFFMT is likely measuring the same face matching abilities as the established GFMT (Burton et al., 2010).

***Comparison between GFMT and SFFMT***

Although human performance on the SFFMT was correlated with the GFMT, our data also suggest that the SFFMT is a harder task. The average overall accuracy of the control participants for the SFFMT’s unfamiliar faces was just 70.1% (SD = 9.3), a decrease of 12.3% from their performance on the GFMT (*M* = 82.4%, *SD* = 11.4). The similarity ratings from the DNN in the main text also support this conclusion; on average, the match trials in the SFFMT (M = 73.8, SD = 6.9) were less similar than those in the GFMT (M = 83.4, SD = 5.9), whereas the mismatch trials in the SFFMT were more similar (M = 21.2, SD = 8.7) than those in the GFMT (M = 9.9, SD = 8.2). These results indicate that the SFFMT is a challenging test of face matching ability. We plan to continue the development of the SFFMT as a test of face matching ability in future.

***Discussion***

Although accuracy was generally impaired for the mixed and masked conditions, Figure S1 shows that the effect of face masks on accuracy varied for match and mismatch trials. For match trials, accuracy was consistently lowest for the masked condition, whereas it was the mixed condition that had the lowest accuracy on mismatched trials. This decrease in accuracy can be substantial, with a 19% decline for the mixed and masked conditions on the GFMT’s match trials, while decreases of 24% and 17% occurred for the mixed and masked conditions on the SFFMT’s familiar mismatch trials. However, the true effect that face masks have on accuracy is obscured by the response biases shown by participants. As reported in the main text, participants in the mixed and masked conditions of the GFMT showed a bias to declare all pairs as “mismatches”, whereas those in the control condition had no bias. A tendency to declare all pairs mismatches will inflate the accuracy of the mixed and masked conditions for mismatched trials; likely explaining why mask condition did not affect accuracy for mismatch trials in the GFMT (see main text for a comprehensive discussion of response biases).

**2. Commercial Deep Neural Networks**

In May 2020, we tested the performance of three commercially available face recognition systems (which are also DNNs) to investigate whether the performance of the research DNN from the main text is typical of commercially available systems. These additional face recognition systems were from Amazon (“Rekognition”), Face ++, and Microsoft (“Face”, Model 2). Because our aim was not to critique these systems individually, we randomly assigned these systems the labels DNN2, DNN3, and DNN4, which are used throughout the supplementary materials. DNN1 refers to the research DNN from the main text. We have plotted DNN1 on the figures throughout this supplemental section, to help highlight similar patterns of performance between the various systems. The commercial DNNs were tested on the same GFMT and SFFMT tasks from the main study. All systems were tested using the default thresholds recommended by the manufacturers.

***Glasgow Face Matching Test***

**Classification Accuracy.** The accuracy of DNN2 was comparable to that of DNN1, with no errors made for the control condition, two errors for mixed stimuli, and three errors for masked stimuli (see Figure S2). Notably, DNN3 often returned ‘no face found’ errors for faces wearing masks, which resulted in missing data (DNN3 was the only system to have this issue). The figures throughout this supplementary results section show the accuracy of DNN3 for the trials where it found both faces in each pair and produced a classification decision^[[1]](#footnote-2)^. When it found a face, the accuracy of DNN3 was also similar to that of DNN1 and DNN2. Finally, DNN4 was generally less accurate than the other systems, but particularly so for mismatch trials. DNN4 only correctly rejected 65% of mismatches for the control stimuli. For masked mismatches, DNN4 failed to correctly reject a single pair. This pattern of results suggests that DNN4’s threshold to declare a match is too liberal (it is biased to declare ‘match’).


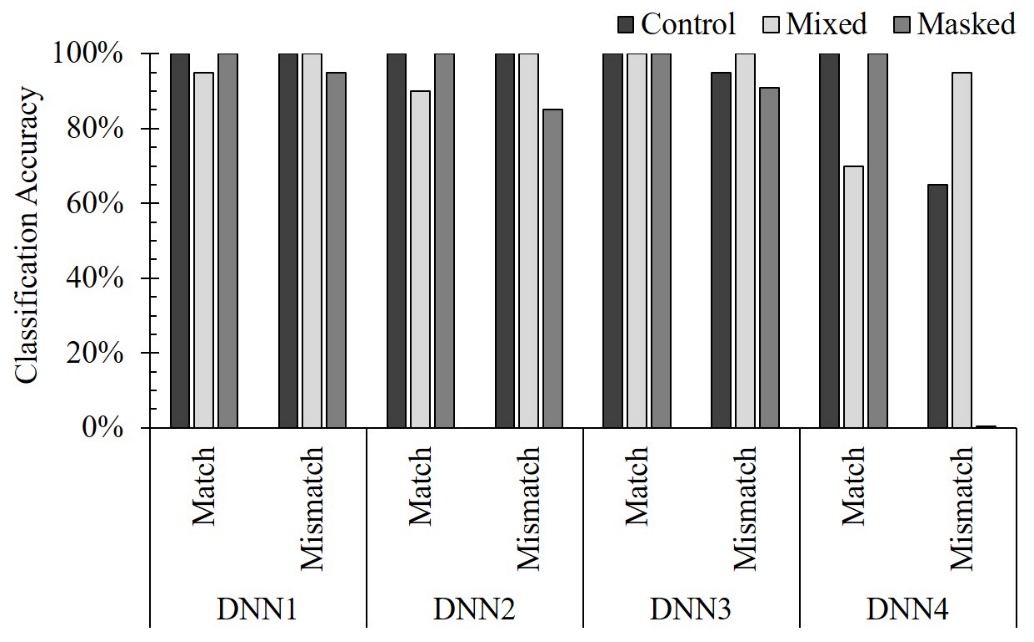


**Figure S2.** The classification accuracy for each DNN on the GFMT, shown as the percentage correct of the 20 trials in each condition, plotted separately for match and mismatch pairs.

**Similarity Ratings.** The descriptive statistics for the similarity ratings made by each commercial DNN are shown in Table S5. The similarity ratings given to the pairs in the GFMT followed a similar pattern across the three commercial DNNs (see Figure S3). Please note, any difference between the absolute similarity ratings given by the different DNN systems is not relevant; they simply reflect the different thresholds that each system uses. For match pairs, all 3 commercial DNNs gave lower similarity ratings to the mixed condition than to the control and masked conditions, which did not differ from each other (see Table S6). These patterns of results are similar to DNN1; however, DNN1 also found the control condition to be more similar than the masked condition. For mismatched pairs, all commercial DNNs gave higher similarity ratings to the masked condition than to the control or mixed conditions. This pattern differs from DNN1, which did not rate the masked condition to be more similar than the other conditions. Only DNN4 gave significantly higher similarity ratings to the control condition than the mixed condition.

**
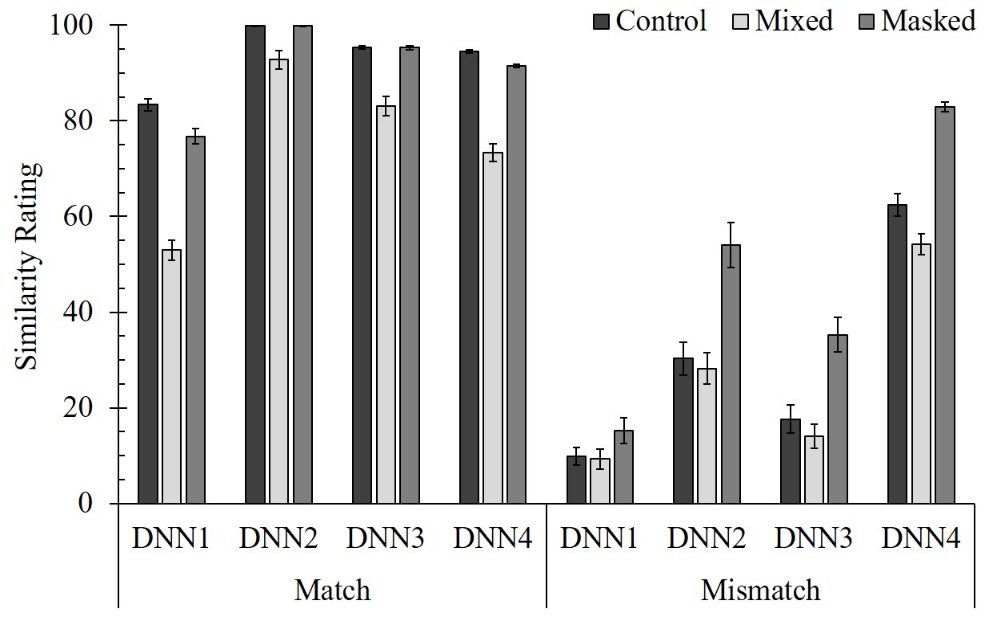
**

**Figure S3.** Similarity ratings from each DNN for the GFMT, plotted separately for match and mismatch trials. Error bars show SEM across items.

**Table S5**

*Descriptive statistics [mean(SD)] for the similarity ratings made by each commercial DNN for the match and mismatch trials of the GFMT.*

|  | **Match** | | | **Mismatch** | | |
| --- | --- | --- | --- | --- | --- | --- |
|  | Control | Mixed | Masked | Control | Mixed | Masked |
| **DNN2** | 99.89 (0.19) | 92.82 (8.63) | 99.85 (0.18) | 30.31 (15.50) | 28.20 (14.61) | 53.97 (21.10) |
| **DNN3** | 95.30 (1.38) | 83.08 (7.19) | 95.29 (1.11) | 17.65 (12.91) | 14.07 (9.82) | 35.27 (12.03) |
| **DNN4** | 94.52 (1.49) | 73.35 (7.90) | 91.45 (1.63) | 62.43 (10.18) | 54.18 (9.77) | 82.93 (4.27) |

**Table S6**

*Separate items-analysis ANOVAs and post-hoc analyses for the similarity ratings made by each DNN for match and mismatch trials of the GFMT. *identifies statistically significant t-tests.*

|  | **Match Pairs** | | | | **Mismatch Pairs** | | | |
| --- | --- | --- | --- | --- | --- | --- | --- | --- |
| **DNN2** | |  |  |  |  |  |  |  |
| Mask Condition | *F*(2, 33.68) = 6.75, *p* = .003, $\text{η}_{\text{p}}^{\text{2}}$ = .32 | | | | *F*(2, 57) = 13.67, *p* < .001, $\text{η}_{\text{p}}^{\text{2}}$ = .32 | | | |
|  | ***t*** | **95%CI** | ***p_bonf_*** | ***d*** | ***t*** | **95%CI** | ***p_bonf_*** | ***d*** |
| Control-Mixed | 4.48 | 3.28, 10.86 | < .001* | 1.16 | 0.39 | -11.06, 15.28 | .999 | 0.14 |
| Control-Masked | 0.02 | -3.76, 3.83 | .999 | 0.21 | -4.32 | -36.84, -10.50 | < .001* | -1.28 |
| Mixed-Masked | -4.46 | -10.83, -3.24 | < .001* | 1.15 | -4.71 | -38.95, -12.60 | < .001* | -1.42 |
|  |  | | | |  | | | |
| **DNN3** | |  |  |  |  |  |  |  |
| Mask Condition | *F*(2, 16.75) = 17.76, *p* < .001, $\text{η}_{\text{p}}^{\text{2}}$ = .66 | | | | *F*(2, 43) = 11.47, *p* < .001, $\text{η}_{\text{p}}^{\text{2}}$ = .35 | | | |
|  | ***t*** | **95%CI** | ***p_bonf_*** | ***d*** | ***t*** | **95%CI** | ***p_bonf_*** | ***d*** |
| Control-Mixed | 8.10 | 8.54, 15.91 | < .001* | 2.66 | 0.89 | -6.18, 13.35 | .999 | 0.31 |
| Control-Masked | 0.01 | -4.53, 4.56 | .999 | 0.01 | -3.99 | -28.35, -6.89 | < .001* | -1.40 |
| Mixed-Masked | -6.15 | -17.06, -7.36 | < .001* | 2.07 | -4.54 | -32.55, -9.86 | < .001* | -1.96 |
|  |  | | | |  | | | |
| **DNN4** | |  |  |  |  |  |  |  |
| Mask Condition | *F*(2, 34.11) = 79.17, *p* < .001, $\text{η}_{\text{p}}^{\text{2}}$ = .80 | | | | *F*(2, 32.25) = 92.00, *p* < .001, $\text{η}_{\text{p}}^{\text{2}}$ = .68 | | | |
|  | ***t*** | **95%CI** | ***p_bonf_*** | ***d*** | ***t*** | **95%CI** | ***p_bonf_*** | ***d*** |
| Control-Mixed | 14.14 | 17.57, 24.78 | < .001* | 3.72 | 3.06 | 1.77, 14.72 | .010* | 0.83 |
| Control-Masked | 2.05 | -0.53, 6.68 | .135 | 1.96 | -7.62 | -26.98, -14.02 | < .001* | -2.63 |
| Mixed-Masked | -12.09 | -21.71, -14.50 | < .001* | -3.17 | -10.68 | -35.22, -22.27 | < .001* | -3.81 |

**Stirling Famous Face Matching Task**

**Classification Accuracy.** For match pairs, each DNN showed high levels of accuracy for control and masked faces, making no more than one error in any condition (see Figure S4a). Like DNN1, DNN2 and DNN4 both showed reduced accuracy for faces in the mixed condition, which indicates that many systems might struggle to correctly match faces when one is covered by a mask and the other is not. DNN3 correctly classified all matching trials that it found a face^[[2]](#footnote-3)^.

For mismatched pairs, DNN2 and DNN3 showed high levels of accuracy in all mask conditions, correctly classifying at least 80% of trials^[[3]](#footnote-4)^. While this level of accuracy is similar to DNN1, DNN2 and DNN3 make slightly more errors. DNN4 again showed very poor performance in the masked condition, correctly rejecting only 1/40 mismatched pairs (see Figure S4b).


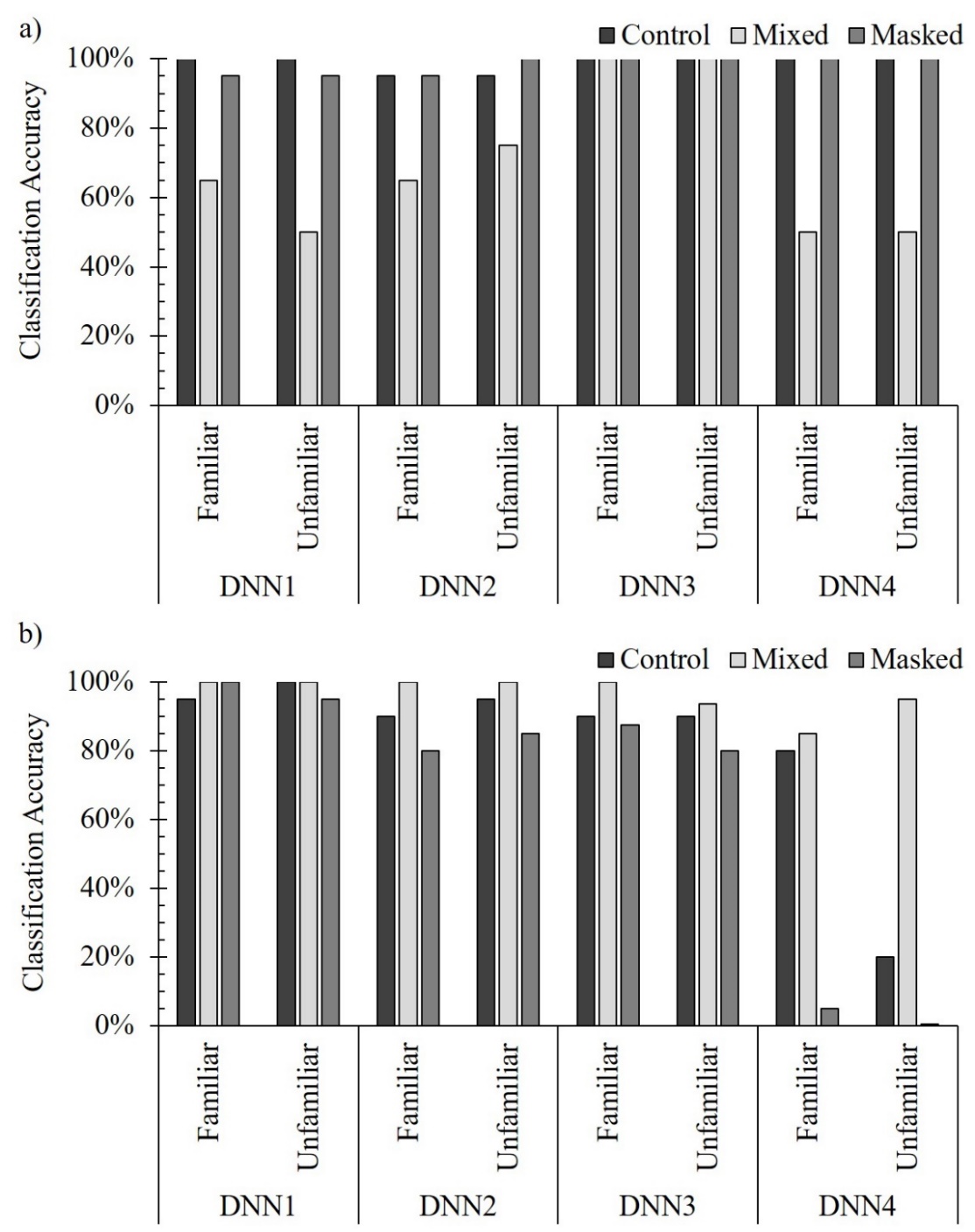


**Figure S4.** The classification accuracy of each DNN for **a)** match trials **b)** mismatch trials in the SFFMT, shown as the percentage correct of the 20 trials in each condition.

**Similarity Ratings.** The descriptive statistics for the similarity ratings made by each commercial DNN are shown in Table S7. Visual inspection of Figure S5a shows that all DNN systems produced comparable patterns of similarity ratings for match trials. Similarity ratings were lower for the mixed condition than for the control and masked conditions (see Table S8). Ratings were significantly higher for the control condition than the masked condition for DNN4, but not DNN2 or DNN3. The main effect of familiarity was non-significant for all DNNs, as was the interaction between mask condition and familiarity. These patterns of results replicate those reported for DNN1 in the main text.

Visual inspection of Figure S5b also reveals a consistent pattern in similarity ratings from the DNNs for mismatched pairs of faces. Once again, the DNNs rated the mixed condition to be less similar than the control and masked conditions (see Table S8). Similarity ratings for the control and masked conditions did not differ for DNN2 or DNN3. DNN4 gave higher similarity ratings to the masked condition than the control condition. The main effect of familiarity was non-significant for all DNNs, as was the interaction between mask condition and familiarity.

**Table S7**

*Descriptive statistics [mean(SD)] for the similarity ratings made by each commercial DNN for the match and mismatch trials of the SFFMT.*

|  | **Match** | | | **Mismatch** | | |
| --- | --- | --- | --- | --- | --- | --- |
|  | Control | Mixed | Masked | Control | Mixed | Masked |
| **DNN2** |  |  |  |  |  |  |
| Unfamiliar | 95.70 (16.99) | 84.33 (14.63) | 97.66 (2.54) | 50.88 (21.68) | 32.23 (18.59) | 51.03 (24.16) |
| Familiar | 94.97 (19.84) | 81.19 (19.73) | 94.43 (17.62) | 45.22 (19.95) | 30.66 (17.90) | 56.64 (23.19) |
| **DNN3** |  |  |  |  |  |  |
| Unfamiliar | 91.88 (1.25) | 69.50 (11.06) | 87.25 (5.92) | 32.50 (15.45) | 17.40 (7.98) | 31.20 (16.12) |
| Familiar | 92.25 (1.71) | 75.00 (12.33) | 89.00 (3.46) | 17.88 (7.32) | 11.88 (4.79) | 29.50 (15.96) |
| **DNN4** |  |  |  |  |  |  |
| Unfamiliar | 91.02 (2.44) | 69.87 (7.32) | 88.29 (2.22) | 72.67 (7.77) | 58.42 (8.89) | 81.84 (3.56) |
| Familiar | 92.22 (2.30) | 69.80 (7.12) | 89.21 (1.95) | 66.35 (7.75) | 55.39 (11.32) | 80.86 (5.65) |


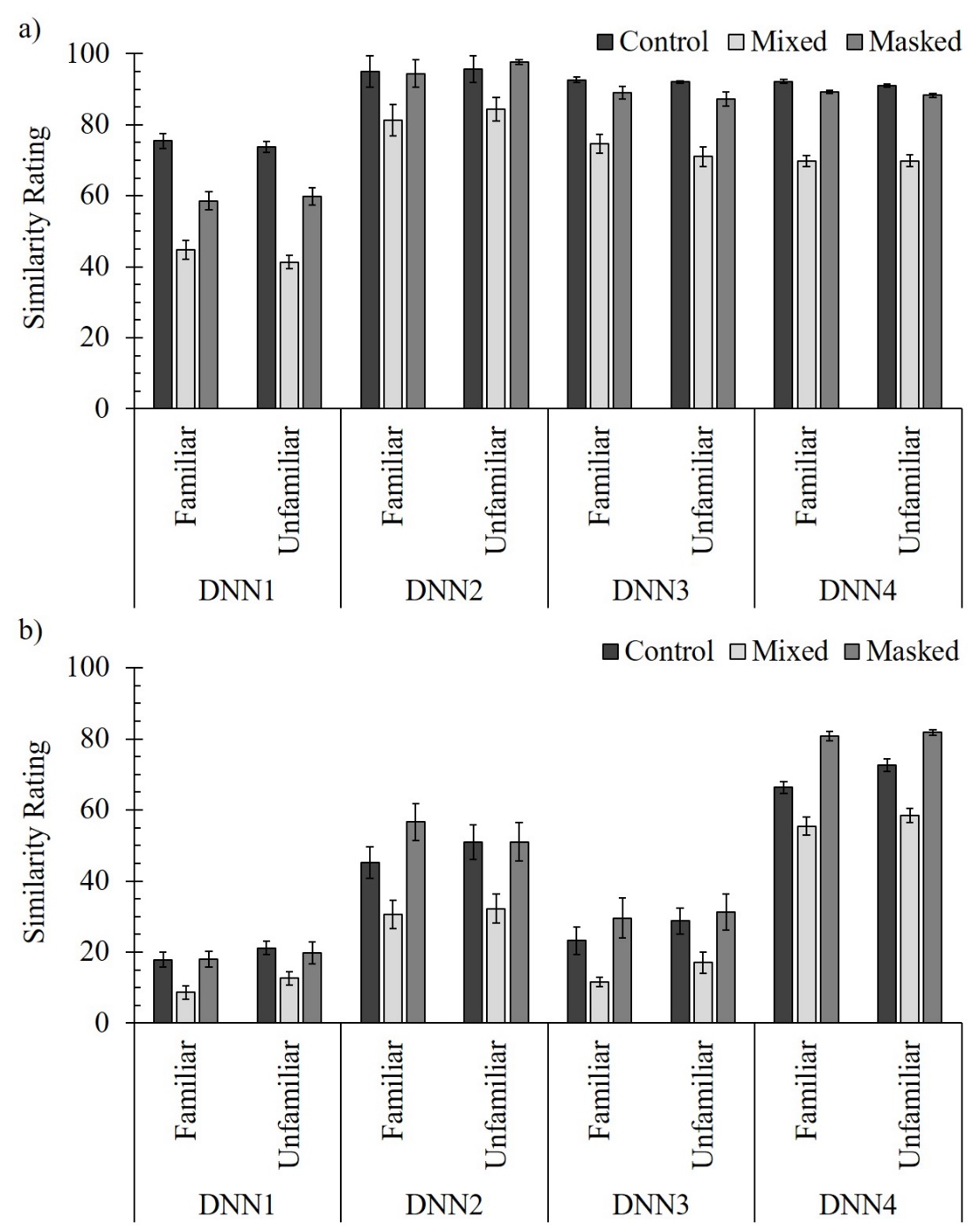


**Figure S5.** Similarity ratings for **a)** matched pairs **b)** mismatched pairs in the SFFMT, plotted separately for familiar and unfamiliar faces. Error bars show SEM across items.

**Table S8**

*Separate item-analysis ANOVAs and post-hoc analyses for the similarity ratings given by the DNNs for match and mismatch trials of the SFFMT. *identifies statistically significant t-tests.*

|  | **Match Pairs** | | | | **Mismatch Pairs** | | | |
| --- | --- | --- | --- | --- | --- | --- | --- | --- |
| **DNN2** |  | | | |  | | | |
| *ANOVA* |  | | | |  | | | |
| Mask Condition | *F*(1.69, 64.12) = 26.65, *p* < .001, $\text{η}_{\text{p}}^{\text{2}}$ = .41 | | | | *F*(2, 76) = 42.45, *p* < .001, $\text{η}_{\text{p}}^{\text{2}}$ = .53 | | | |
| Familiarity | *F*(1, 38) = 0.27, *p* = .609, $\text{η}_{\text{p}}^{\text{2}}$ = .01 | | | | *F*(1, 38) = 0.01, *p* = .929, $\text{η}_{\text{p}}^{\text{2}}$ = .00 | | | |
| Interaction | *F*(1.69, 64.12) = 0.24, *p* = .750, $\text{η}_{\text{p}}^{\text{2}}$ = .01 | | | | *F*(2, 76) = 2.56, *p* = .084, $\text{η}_{\text{p}}^{\text{2}}$ = .06 | | | |
|  |  | | | |  | | | |
| *Mask Condition* | ***t*** | **95%CI** | ***p_bonf_*** | ***d*** | ***t*** | **95%CI** | ***p_bonf_*** | ***d*** |
| Control-Mixed | 6.14 | 7.56, 17.59 | < .001* | 0.97 | 6.58 | 10.43, 22.79 | < .001* | 1.04 |
| Control-Masked | -0.35 | -5.73, 4.30 | .999 | -0.06 | -2.29 | -11.96, 0.39 | .074 | -0.36 |
| Mixed-Masked | -6.49 | -18.30, -8.28 | < .001* | -1.03 | -8.88 | -28.57, -16.22 | < .001* | -1.40 |
|  |  | | | |  | | | |
| **DNN3** |  | | | |  | | | |
| *ANOVA* |  | | | |  | | | |
| Mask Condition | *F*(1.11, 11.11) = 34.81, *p* < .001, $\text{η}_{\text{p}}^{\text{2}}$ = .78 | | | | *F*(2, 32) = 13.73, *p* < .001, $\text{η}_{\text{p}}^{\text{2}}$ = .46 | | | |
| Familiarity | *F*(1, 10) = 0.55, *p* = .475, $\text{η}_{\text{p}}^{\text{2}}$ = .05 | | | | *F*(1, 16) = 2.40, *p* = .141, $\text{η}_{\text{p}}^{\text{2}}$ = .13 | | | |
| Interaction | *F*(1.11, 11.11) = 0.56, *p* = .489, $\text{η}_{\text{p}}^{\text{2}}$ = .05 | | | | *F*(2, 32) = 2.36, *p* = .111, $\text{η}_{\text{p}}^{\text{2}}$ = .13 | | | |
|  |  | | | |  | | | |
| *Mask Condition* | ***t*** | **95%CI** | ***p_bonf_*** | ***d*** | ***t*** | **95%CI** | ***p_bonf_*** | ***d*** |
| Control-Mixed | 7.88 | 13.24, 26.38 | < .001* | 2.28 | 3.45 | 2.83, 18.27 | .005* | 0.81 |
| Control-Masked | 1.57 | -2.63, 10.51 | .399 | 0.45 | -1.69 | -12.88, 2.56 | .303 | -0.40 |
| Mixed-Masked | -6.31 | -22.44, -9.31 | < .001* | -1.82 | -5.14 | -23.43, -7.99 | < .001* | -1.21 |
|  |  | | | |  | | | |
| **DNN4** |  | | | |  | | | |
| *ANOVA* |  | | | |  | | | |
| Mask Condition | *F*(1.19, 45.12) = 357.03, *p* < .001, $\text{η}_{\text{p}}^{\text{2}}$ = .90 | | | | *F*(1.71, 64.91) = 162.89, *p* < .001, $\text{η}_{\text{p}}^{\text{2}}$ = .81 | | | |
| Familiarity | *F*(1, 38) = 0.46, *p* = .502, $\text{η}_{\text{p}}^{\text{2}}$ = .01 | | | | *F*(1, 38) = 3.16, *p* = .084, $\text{η}_{\text{p}}^{\text{2}}$ = .08 | | | |
| Interaction | *F*(1.19, 45.12) = 0.29, *p* = .635, $\text{η}_{\text{p}}^{\text{2}}$ = .01 | | | | *F*(1.71, 64.91) = 1.98, *p* = .153, $\text{η}_{\text{p}}^{\text{2}}$ = .05 | | | |
|  |  | | | |  | | | |
| *Mask Condition* | ***t*** | **95%CI** | ***p_bonf_*** | ***d*** | ***t*** | **95%CI** | ***p_bonf_*** | ***d*** |
| Control-Mixed | 24.59 | 19.62, 23.95 | < .001* | 3.89 | 9.31 | 9.29, 15.92 | < .001* | 1.47 |
| Control-Masked | 3.24 | 0.70, 5.04 | .005* | 0.51 | -8.74 | -15.15, -8.52 | < .001* | -1.38 |
| Mixed-Masked | -21.35 | -21.08, -16.75 | < .001* | -3.38 | -18.05 | -27.75, -21.12 | < .001* | -2.85 |

***Discussion***

Our aim in conducting these additional analyses was to investigate whether the high performance of the naïve research DNN (DNN1) for faces occluded by surgical masks was typical of commercially available face recognition systems (to the best of our knowledge, these systems had also not been trained to identify masked faces). In general, DNN2 performed similarly to DNN1, both in terms of classification accuracy, and in the pattern of similarity ratings that it gave to the faces in different mask conditions. However, testing DNN3 and DNN4 revealed two issues that might arise from using naïve face recognition systems to verify the identity of faces that are wearing surgical masks.

DNN3 had trouble locating faces that were occluded by surgical face masks (the issue is clearly related to the surgical masks, because there were fewer missing data points for the mixed condition than the masked condition). This issue was most pronounced in the SFFMT, where the system only found both faces on 12/40 match trials for the masked condition. As we note throughout the results section, DNN3 showed comparable performance to DNN1 and DNN2, when it was able to locate both faces in the pair to produce a classification. One possibility is that DNN3 located faces on the easier trials, which would contribute to its high levels of performance. Nonetheless, these findings demonstrate that one issue with using naïve DNNs to match masked faces is simply that they might not be able to find the occluded face in the image at all.

At the other end of the continuum, DNN4 had no issue finding faces that were occluded by surgical face masks, but it declared nearly all pairs that both faces wore masks to be “matches”. Although part of this issue relates to the threshold used by DNN4^[[4]](#footnote-5)^, the similarity ratings from DNNs 2-4 reveal a more pervasive issue. For mismatch pairs, the commercial DNNs all tended to rate the faces in the masked condition as being more similar than the control stimuli. This pattern of results strongly suggests that the three commercial DNNs were actively matching the masks on each face to each other. As a result, these systems are liable to produce false positive classifications for mismatched faces, when both faces are wearing surgical masks. The most dramatic example of this issue can be seen in DNN4, which incorrectly classified 39/40 mismatched pairs as being “matches” when both faces were wearing masks. Future research must be directed toward preventing the DNNs from incorporating non-face occlusions, such as surgical masks, into their evaluation of the two faces that are being compared. Our findings clearly demonstrate that high levels of caution must be exercised when evaluating the classification decisions made by naïve DNNs for masked faces, because they may be biased toward producing false positive classifications.

**Supplementary References**

Burton, A. M., White, D., & McNeill, A. (2010). The Glasgow Face Matching Test. *Behavior Research Methods, 42*(1), 286-291. doi:10.3758/brm.42.1.286

Megreya, A. M., & Burton, A. M. (2007). Hits and false positives in face matching: A familiarity-based dissociation. *Perception & Psychophysics, 69*(7), 1175-1184. doi:10.3758/bf03193954

Noyes, E., & Jenkins, R. (2019). Deliberate disguise in face identification. *Journal of Experimental Psychology: Applied, 25*(2), 280-290. doi:10.1037/xap0000213

1. DNN3 only found both faces on 28/40 mixed condition trials, and just 18/40 trials in the masked condition. If we were to count trials that DNN3 could not find a face as errors, classification accuracy in the mixed condition would fall to 65% for match trials and 75% for mismatch trials, while classification accuracy in the masked condition would fall to 35% for match trials and 50% for mismatch trials. [↑](#footnote-ref-2)
2. For matching pairs, DNN3 only found 26/40 pairs of faces in the mixed condition (20 familiar pairs, 20 unfamiliar pairs), and just 12/40 pairs for masked condition. If the trials that DNN3 could not find a face were counted as errors, classification accuracy in the mixed condition would fall to 60% for familiar pairs and 70% for unfamiliar pairs, while accuracy in the masked condition would fall to 20% for familiar pairs and 40% for unfamiliar pairs. [↑](#footnote-ref-3)
3. For mismatched pairs, DNN3 only found 26/40 pairs of faces in the mixed condition, and just 18/40 pairs for masked condition. If trials that DNN3 could not find a face were counted as errors, classification accuracy in the mixed condition would fall to 50% for familiar pairs and 75% for unfamiliar pairs, while accuracy in the masked condition would fall to 35% for familiar pairs and 40% for unfamiliar pairs. [↑](#footnote-ref-4)
4. Notably, a lack of accuracy was also seen from DNN4 for mismatched pairs in the control condition, suggesting that part of the issue is that DNN4’s unique threshold is too liberal (since many mismatched pairs are classified as “matches”). [↑](#footnote-ref-5)
